# Siderite-based anaerobic iron cycle driven by autotrophic thermophilic microbial consortium

**DOI:** 10.1038/s41598-020-78605-7

**Published:** 2020-12-10

**Authors:** Daria G. Zavarzina, Tatiana V. Kochetkova, Nataliya I. Chistyakova, Maria A. Gracheva, Angelina V. Antonova, Alexander Yu. Merkel, Anna A. Perevalova, Michail S. Chernov, Yury A. Koksharov, Elizaveta A. Bonch-Osmolovskaya, Sergey N. Gavrilov, Andrey Yu. Bychkov

**Affiliations:** 1 Winogradsky Institute of Microbiology, Federal Research Centre “Fundamentals of Biotechnology”, Russian Academy of Sciences, Prospekt 60 Letiya Oktyabrya 7, bld. 2, Moscow, 117312 Russian Federation; 2 Department of Physics, Lomonosov Moscow State University, Leninskie Gory 1, Moscow, 119991 Russian Federation; 3 Department of Analytical Chemistry, Institute of Chemistry, Eötvös Loránd University, Pázmány P. s. 1/A, Budapest, 1117 Hungary; 4 Department of Geology, Lomonosov Moscow State University, Leninskie Gory 1, Moscow, 119991 Russian Federation; 5 Department of Biology, Lomonosov Moscow State University, Leninskie Gory 1-12, Moscow, 119991 Russian Federation

**Keywords:** Element cycles, Precambrian geology, Biogeochemistry, Microbial communities, Environmental microbiology

## Abstract

Using a sample from a terrestrial hot spring (pH 6.8, 60 °C), we enriched a thermophilic microbial consortium performing anaerobic autotrophic oxidation of hydrothermal siderite (FeCO_3_), with CO_2_/bicarbonate as the electron acceptor and the only carbon source, producing green rust and acetate. In order to reproduce Proterozoic environmental conditions during the deposition of banded iron formation (BIF), we incubated the microbial consortium in a bioreactor that contained an unmixed anoxic layer of siderite, perfectly mixed N_2_/CO_2_-saturated liquid medium and microoxic (2% O_2_) headspace. Long-term incubation (56 days) led to the formation of magnetite (Fe_3_O_4_) instead of green rust as the main product of Fe(II) oxidation, the precipitation of newly formed metabolically induced siderite in the anoxic zone, and the deposition of hematite (Fe_2_O_3_) on bioreactor walls over the oxycline boundary. Acetate was the only metabolic product of CO_2_/bicarbonate reduction. Thus, we have demonstrated the ability of autotrophic thermophilic microbial consortium to perform a short cycle of iron minerals transformation: siderite–magnetite–siderite, accompanied by magnetite and hematite accumulation. This cycle is believed to have driven the evolution of the early biosphere, leading to primary biomass production and deposition of the main iron mineral association of BIF.

## Introduction

Siderite (FeCO_3_) is one of the main ferrous-containing minerals in the Earth’s crust that is involved in the global cycling of iron and carbon. A significant part of existing siderite was accumulated in banded iron formations (BIF), known to be the largest source of iron ores in the world^1–4^. These sedimentary marine rocks consisting of Fe-rich laminae (bands) containing magnetite, martite, hematite, siderite, iron silicates and, to a lesser extent, pyrite alternating with cherty Si-rich layers, formed on all continents during a period lasting from 3800 to 1800 million years ago^4,5^. Most researchers agree that iron at the time existed in its ferrous form, and some suggest that siderite was the primary mineral in these formations^2,5–8^. It is also generally accepted that microorganisms such as phototrophs, photoferrotrophs, nitrate-dependent iron oxidisers, and iron reducers participated in BIF deposition^3–5,9–11^. Thus, siderite is one of the minerals that could have been directly involved in biogeochemical cycles that were functional long before the appearance of oxygen in the atmosphere^2,5,6,8^.

Iron can serve as an energy source or electron acceptor for many prokaryotes and is the second most abundant redox-active element on Earth, after oxygen^9^. The discovery of dissimilatory iron-reducing bacteria (DIRB)^12,13^ prompted active investigation of the ability of these microorganisms to transform Fe(III)-containing minerals. Dissimilatory Fe(III)-reducing prokaryotes inhabit almost all mineral-rich anoxic environments^14–18^ and gain energy through the reduction of various Fe(III)-containing minerals, e.g. oxyhydroxides^19,20^, clay minerals^21,22^, and phylosilicates^23^. It is worth noting that magnetite and siderite are the main reduced minerals produced in microbial iron reduction^14–17^.

Anaerobic microbial oxidation of ferrous iron has not been the subject of a significant amount of research. Two processes have been studied the most, photosynthetic Fe(II)-oxidation^24^ and nitrate-dependent Fe(II)-oxidation^25^, and several researchers have demonstrated the possibility of siderite oxidation in both of them^24–26^. Both of these processes, which are thought to be involved in BIF deposition^3,4,9–11^, would have had limited energy sources under the Precambrian conditions. Photoferrotrophs require specific surface habitats with access to both light and ferrous iron, while nitrate-dependent Fe(II)-oxidising bacteria require the presence of nitrate, which is unlikely to have been available in significant amount under the anoxic conditions of early Earth^3,9,10^. The third, recently demonstrated route of anaerobic microbial iron oxidation has only been shown for alkaliphilic bacteria. The autotrophic Fe(III)-reducing bacterium *Geoalkalibacter* *ferrihydriticus* was shown to be capable of transforming biotite and glauconite, Fe(II)/Fe(III)-containing phyllosilicates, to magnetite in the presence of acetate at pH 9.5^17^. During the experiment, the additional formation of acetate was observed instead of its expected oxidation, while Mӧssbauer spectral analysis revealed that magnetite was formed due to Fe(II)-oxidation rather than Fe(III)-reduction. Based on these observations, we hypothesize that this microorganism would be able to perform carbonate-dependent Fe(II)-oxidation. Thermodynamic calculations supported this suggestion^17^. Further investigations demonstrated the ability of alkaliphilic autotrophic acetogenic bacterium *Fuchsiella* *ferrireducens*, as well as a syntrophic culture of *G.* *ferrihydriticus* and *Candidatus* “Contubernalis alkalaceticum” to oxidise siderite anaerobically in the presence of ethanol^27,28^.

Genomic reconstruction of the last universal common ancestor (LUCA) metabolism performed by Weiss et al. revealed that LUCA was probably an anaerobic, CO_2_-fixing, H_2_-dependent and N_2_-fixing thermophilic microorganism capable of performing the Wood–Ljungdahl pathway^29^. On the other hand, extracellular electron transfer (EET), including processes driving anaerobic reduction and oxidation of iron minerals, are thought to have been amongst the first respiratory pathways that appeared on the hot early Earth^30^. Thus, carbonate-dependent iron oxidation by thermophilic microorganisms may have play a significant role in the early development of Earth biosphere.

The main goal of the present research was to demonstrate the capability of a thermophilic microbial consortium to perform autotrophic anaerobic oxidation of hydrothermal siderite. Cultivating the consortium in a bioreactor with a microoxic headspace, simulating the conditions of early/mid Proterozoic ocean, we were able to assess the possible contribution of this process to BIF accumulation.

## Results

### Obtaining a stable anaerobic thermophilic siderite-oxidising microbial consortium

We obtained the primary enrichment culture from a sediment/water sample of the Solnechny hot spring (‘Sun spring’, the Uzon Caldera, Kamchatka Peninsula, Russia), which has a temperature of at 60 °C and pH of 6.8. A stable siderite-oxidising anaerobic thermophilic microbial consortium was obtained by ten-fold dilutions and multiple consequent transfers (see “Methods”) on bicarbonate medium with hydrothermal siderite as the electron donor and 20% of CO_2_ in the gas phase as the electron acceptor. After ten days of incubation, the population density had reached 5 × 10^6^ cell ml^−1^ and up to 0.5 mM acetate had formed. The initial siderite had been transformed into a grey precipitate (Supplementary Fig. S1) and thin mineral lamellae of green-brownish colour. The new mineral phase was identified by Mössbauer spectroscopy as green rust, FeCO_3_ × 3Fe(OH)_2_ × 2Fe(OH)_3_ × 2H_2_O (Supplementary Fig. S2a,b; Supplementary Table S1). A Mössbauer spectrum of the sterile control showed no changes in the initial mineral phase of hydrothermal siderite (Supplementary Fig. S2c,d). Using scanning electron microscopy (SEM), thin spherical crystals of green rust were found on the surface of hydrothermal siderite particles. In addition, dense sphere-shaped particles with an average size of 2–5 µm were observed to precipitate separately (Supplementary Fig. S3b,c). The lamellae, with thickness ranging from 15 to 20 μm, consisted of tabular crystals of green rust arranged in mutually perpendicular orientation (Supplementary Fig. S3d–f).

The analysis of microbial abundance and diversity in the siderite-oxidising consortium, based on the high throughput sequencing and counting of 16S ribosomal ribonucleic acid (rRNA) gene amplicons, showed predominance of Gram-positive bacteria of the genus *Carboxydothermus* (69% of the total community). The rest of operational taxonomic units (OTUs) belonged to the genera *Fervidobacterium* (11%), *Thermanaerothrix* (6%), *Thermodesulfovibrio* (4%), *Thermoanaerobaculum* and *Paludibaculum* (1% total) (Supplementary Fig. S4).

### Scaling up the anaerobic siderite-oxidising microbial consortium

In order to reproduce possible processes of biogeochemical siderite transformation in Proterozoic environments, the enriched microbial consortium was incubated in a bioreactor. The reactor was set up in a mixing regime that allowed for the separation of three different phases: an unmixed hydrothermal siderite layer at the bottom of the reactor; a water column intermittently bubbled with N_2_/CO_2_ (80/20) gas mixture to saturation; and headspace gas (15% of the total volume) composed of N_2_/CO_2_/O_2_ (78/20/2 v/v). Gas outflow was controlled to prevent both air entrapment and unwanted rise in the amount of dissolved CO_2_ due to overpressure. The experiment lasted 56 days.

**Figure 1:**
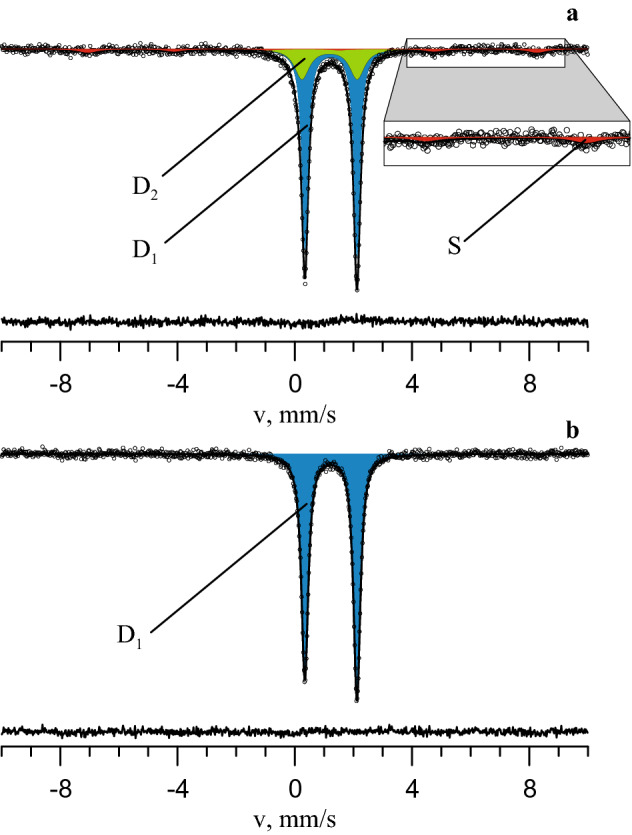
Mössbauer spectrum measured at room temperature of the mineral phase sampled from the bioreactor culture (**a**) and sterile control (**b**) on the 56th day of incubation. D_1_ corresponds to Fe^2+^ ions in the structure of initial siderite, D_2_—to Fe^2+^ ions in the structure of biogenic siderite, S—to magnetically ordered phase.

Unlike with the initial culture, no change of siderite colour was observed in the bioreactor-grown consortium during the course of the experiment. Mössbauer spectroscopic monitoring of siderite transformation revealed a new doublet, steadily increasing over the course of incubation, that was identified as newly formed siderite (Fig. 1a, Table 1, Supplementary Fig. S5, Supplementary Table S2). X-ray powder diffraction analysis (XRD) of a mineral sample taken at the end of the experiment (Supplementary Fig. S6) revealed the presence of the initial hydrothermal siderite (lattice parameters: *a* = 4.684 ± 0.001 Å, *c* = 15.386 ± 0.001 Å) and newly formed siderite (*a* = 4.701 ± 0.001 Å, *c* = 15.407 ± 0.005 Å), thus confirming the results of Mössbauer spectroscopic analysis. In addition to the new doublet, Mössbauer spectra revealed a sextet which was present almost constantly throughout the experiment and could be attributed to a magnetically ordered phase (Fig. 1a, Table 1, Supplementary Fig. S5). Based on its parameters, this magnetically ordered phase was identified as magnetite (Supplementary Table S2). The presence of magnetite was supported by the electron paramagnetic resonance (EPR) spectral characteristics of experimental and control mineral samples determined at the end of incubation. A considerable signal of the magnetic material was observed in the spectra of the experimental samples (Supplementary Fig. S7). Its width was about 1300 Oe and the g-factor was equal to 2.3, supporting that the sample contained magnetite, probably, in the form of nanoparticles^31^.

**Table 1:** Summarized parameters of the bioreactor culture for the 56-day incubation period.

Day	Cell density, cells/ml	Phylogenetic composition of the microbial consortium, %	Acetate, mM	Mineral phase composition, %
0	3.0 × 10^5^	*Carboxydothermus* (69), *Fervidobacterium* (11), *Thermanaerothrix* (6), *Thermodesulfovibrio* (4), *Thermoanaerobaculum* (1), *Paludibaculum* (0.3), *Chthonomonadales* (0.07)	0	HS (100)
7	1.0 × 10^7^	ND	0.01	HS (100)
14	1.1 × 10^7^	*Carboxydothermus* (5), *Fervidobacterium* (0.5), *Thermanaerothrix* (90), *Thermodesulfovibrio* (2), *Thermoanaerobaculum* (0.7), *Paludibaculum* (1), *Chthonomonadales* (0.06)	0.06	HS (91) MIS (5), M (4)
21	0.9 × 10^7^	ND	ND	HS (90), MIS (6), M (4)
28	1.9 × 10^7^	*Carboxydothermus* (0.1), *Fervidobacterium* (0.04), *Thermanaerothrix* (98), *Thermodesulfovibrio* (0.5), *Thermoanaerobaculum* (0.09), *Paludibaculum* (0.5), *Chthonomonadales* (0.1)	0.12	HS (80), MIS (13), M (7)
35	2.5 × 10^7^	ND	ND	HS (79), MIS (15), M (6)
42	7.6 × 10^7^	*Carboxydothermus* (0.3)*,* *Fervidobacterium* (0.5)*,* *Thermanaerothrix* (80)*,* *Thermodesulfovibrio* (9)*,* *Thermoanaerobaculum* (0.1)*,* *Paludibaculum* (5)*,* *Chthonomonadales* (4)*,* *Thermaerobacter* (2)	0.21	HS (79), MIS (17), M (4)
49	2. 6 × 10^7^	ND	0.09	HS (75), MIS (18), M (7)
56	0.9 × 10^7^	*Carboxydothermus* (0.2), *Fervidobacterium* (0.5), *Thermanaerothrix* (79), *Thermodesulfovibrio* (7), *Thermoanaerobaculum* (0.5), *Paludibaculum* (6), *Chthonomonadales* (4), *Thermaerobacter* (3)	0.1	HS (70), MIS (23), M (7)

HS, hydrothermal siderite; MIS, metabolically induced siderite; M, magnetite; ND, not determined.

*E*_*h*_ readings performed in the middle part of water column during the long-term incubation fluctuated from − 7 to − 109 mV [vs standard hydrogen electrode (SHE)] indicating anaerobic conditions. During short periods of active mixing of the culture prior to each sampling (see “Methods”*** section), mineral particles rose to the top part of the bioreactor and precipitated onto its glass walls and rotating shaft. The bottom edge of the precipitate, as well as the results of an independent *E*_*h*_ measurement in the upper part of the liquid medium column at the end of incubation (+ 30 mV vs SHE), indicated oxycline (Supplementary Fig. S8). This mineral precipitate was analysed using Mössbauer spectroscopy and was found to consist of a mixture of hydrothermal siderite and newly formed phases: hematite micro-particles (18.9 ± 0.6%), green rust (17.5 ± 2.8%), and an unidentified phase containing Fe(III) (10.6 ± 0.5%) (Supplementary Fig. S9; Supplementary Table S3). No changes in the initial mineral phase of siderite were observed by Mössbauer spectroscopy in concurrently incubated sterile controls (Fig. 1b) despite of 2% O_2_ present in their headspace. No soluble or HCl-extractable ferrous or ferric iron species were detected colorimetrically in the bioreactor culture or the control at any time during the incubation. The pH of the bioreactor culture was sustained automatically, and no changes in the pH of both culture and uninoculated control were detected during incubation, indicating that iron speciation in the studied media was not influenced by changes in proton activity.

**Figure 2:**
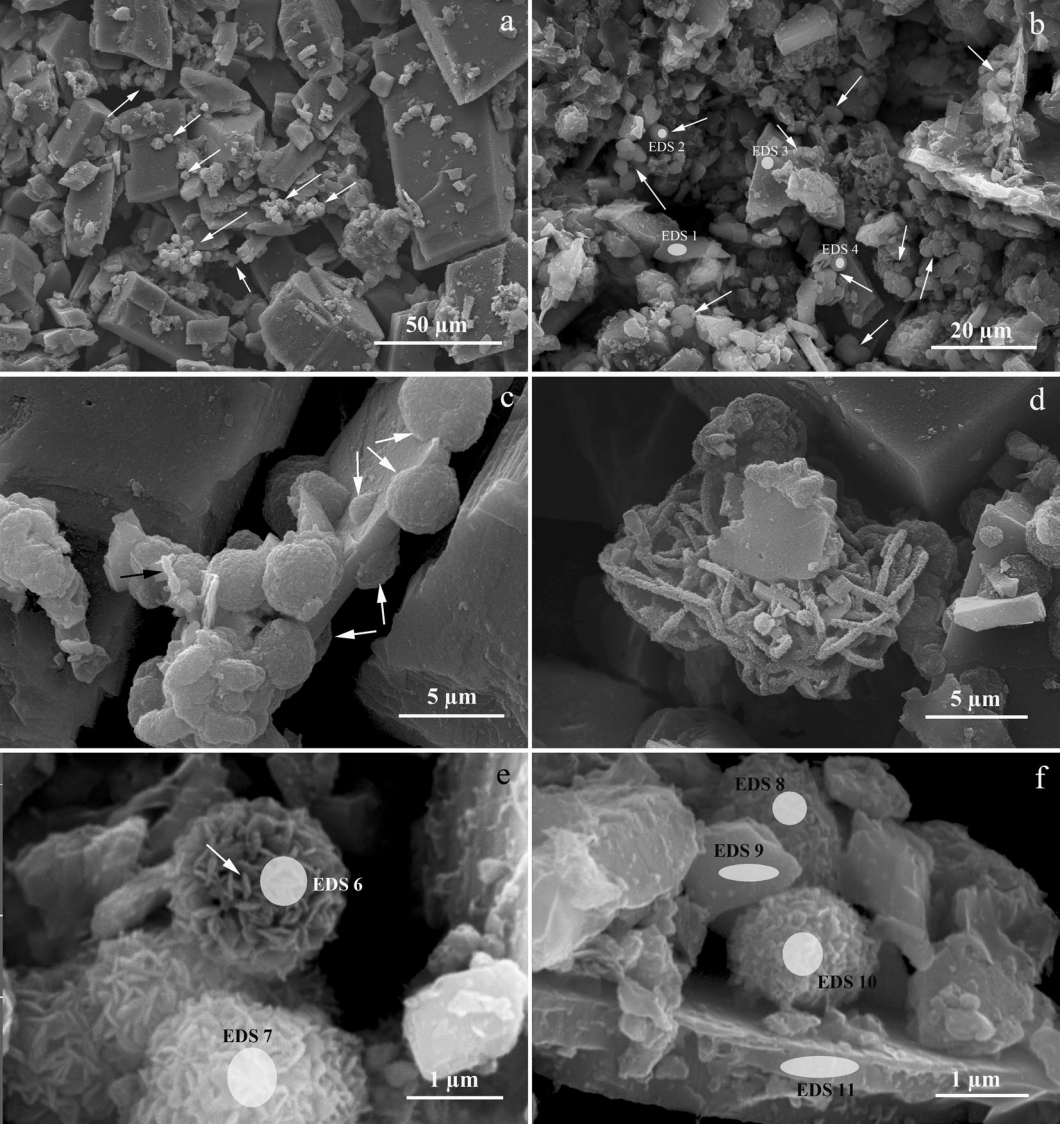
SEM micrographs of the mineral phase (bioreactor culture) sampled after the end of the experiment: (**a**) general view of hydrothermal siderite particles with newly formed globules pointed by white arrows; (**b**) hydrothermal siderite and newly formed globules (white arrows); spots of EDS analysis (white ovals) (Supplementary Table S4); (**c**) metabolically induced siderite globules formed on the surface of hydrothermal siderite (white arrows) and bacterial cells (black arrow); (**d**) fossilized bacterial cells; (**e**,**f**) structure of globules consisting of small flat crystals. White ovals indicate spots of EDS analysis (Supplementary Table S4).

**Figure 3:**
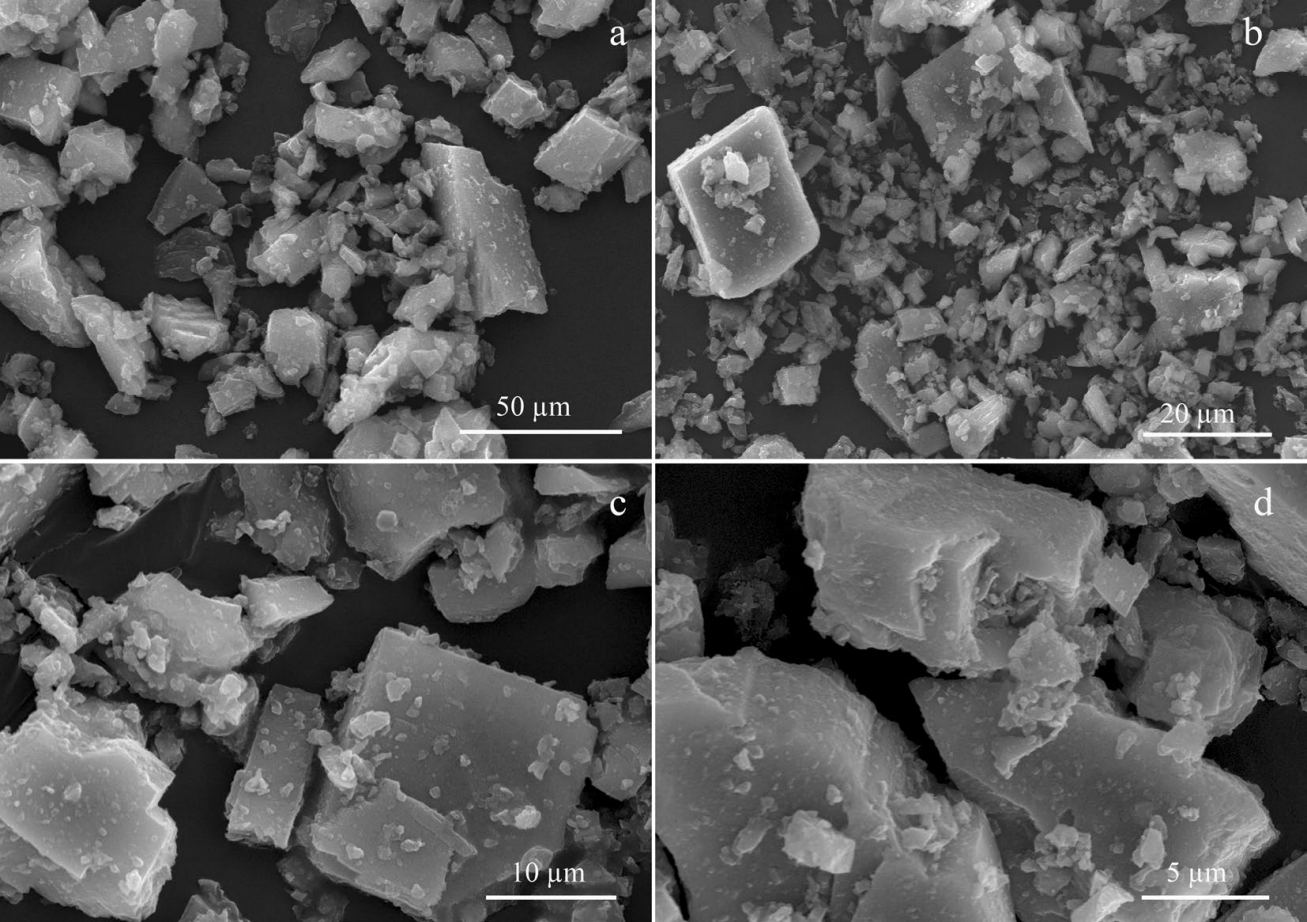
SEM micrographs of hydrothermal siderite particles from sterile control after the end of incubation at different (**a**–**d**) magnifications.

At the end of the experiment, SEM revealed abundant spherical-shaped particles in the mineral phase of the bioreactor (globules Fig. 2a,b), with an average size of about 2–5 µm, typical of previously reported metabolically induced siderite^32^. Some globules were formed on the surface of the initial hydrothermal siderite particles (Fig. 2c). Mineral nanoparticles encrusting bacterial cells were also observed (Fig. 2c,d). The globules consisted of small nanometric-size, slightly curved flat crystals (Fig. 2e,f). Energy dispersive X-ray spectroscopy (EDS) revealed that the elemental composition of the newly formed globules was identical to that of the particles of hydrothermal siderite and, thus, supported the data from the Mössbauer spectroscopy and XRD. The only significant difference in elemental composition of the hydrothermal siderite and globules was the increased content of phosphorus in the globules that indicated their biogenic origin (Fig. 2b,e,f; Supplementary Table S4). SEM/EDS studies revealed no significant changes of hydrothermal siderite in sterile control (Fig. 3).

16S rRNA gene fragment analysis showed the predominance of bacteria of the genus *Thermanaerothrix* in the bioreactor from the 25th day of incubation until the end of the experiment (Table 1). Total cell density increased from 3.0 × 10^5^ to 1.0 × 10^7^ cell ml^−1^ by the 7th day of the experiment and then varied only slightly, as did the acetate concentration (Table 1).

Total organic carbon (TOC) content in the mineral phases was analysed using Rock–Eval pyrolysis method^33^ at the end of incubation and was twenty times higher in the experimental sample than in the control (0.043 versus 0.002 wt%) (Supplementary Table S5). This result clearly indicated active carbon assimilation by the microbial consortium during its growth in the bioreactor.

## Discussion

Thermophilic prokaryotes are believed to originate around 3.8 billion years ago, when the Earth surface had cooled down enough for stable atmosphere and ocean to form; however, ocean temperatures were still elevated, ranging from 55 to 80 °C^34^. A wide variety of thermophilic bacteria and archaea inhabit modern thermal environments, many of which (especially those of volcanic origin) retain physical and chemical conditions of early Earth. For our experiments, we chose a terrestrial hot spring Solnechny in the Uzon Caldera (Kamchatka volcanic belt), previously shown to have a highly diverse microbial community and visible deposition of iron minerals^35^. Using the ten-fold dilution method and consequent transfers on the medium with hydrothermal siderite as a sole electron donor, we obtained a consortium of thermophilic bacteria able to derive energy from iron redox transformation (Supplementary Fig. S4). Bacteria of the genus *Carboxydothermus*, which were present in the enrichment are perfect candidates for performing carbonate-dependent iron oxidation, as some of them possess both the Wood-Ljungdahl and EET pathways^36–38^. Species of other genera, e.g., *Thermodesulfovibrio,* *Thermoanaerobaculum* and *Paludibaculum,* are capable of iron reduction^39–41^. The ability of these organisms to carry out anaerobic Fe(II) oxidation has never been tested. However, the proposed phylogenetic relations between Fe(III)-reducing and Fe(II)-oxidising EET pathways^38,42^ as well as the demonstration of the ability of *G.* *ferrireducens* and *F.* *ferrireducens* to reduce and oxidise iron minerals under anoxic conditions^17,27,32^ support such a possibility.

Mössbauer spectroscopy identified green rust, which is a mixed-valent Fe(II)–Fe(III) hydroxycarbonate, as the sole product of microbial siderite transformation by the initial consortium. Usually, green rust is an intermediate product of aerobic oxidation of ferrous hydroxide, anaerobic iron transformation in hydromorphic soils^43^ or anaerobic ferric oxihydroxides and magnetite reduction by DIRB^41^. On these grounds, green rust is assumed to play an important role in the biogeochemical cycle of iron; it could be found under environmental conditions that equally favour biogenic iron oxidation and iron reduction^45^. In addition to green rust, SEM analysis revealed the formation of new spherical particles (Supplementary Fig. S3b,c) which we assumed to represent a metabolically induced siderite. However, the Mössbauer spectra of siderite and subspectra of Fe(II)-positions in green rust are too similar for these minerals to be distinguished.

The incubation of the siderite-oxidising microbial consortium in a bioreactor with two different mixing modes and 2% O_2_ in the headspace was designed to simulate Proterozoic environmental conditions^46^. This experiment confirmed biogenic redox cycling from hydrothermal to metabolically induced siderite, proposed to occur in the initial enrichment. The conditions reproduced in the bioreactor, specifically the maintenance of a constant CO_2_ concentration in the liquid medium and the presence of a microoxic zone near the gas-to-liquid interface, significantly influenced both the phylogenetic composition of the consortium (Table 1) and the products of siderite transformation (Figs. 1, 2; Table 1; Supplementary Fig. S5; Supplementary Table S2). For example, bacteria of the genus *Thermaerobacter* capable of aerobic growth appeared amongst the obligate anaerobes.

Two new mineral phases, magnetite and newly formed siderite, were detected in the unmixed mineral phase of the bioreactor culture by Mössbauer and EPR spectroscopy, XRD and SEM/EDS. Thermodynamic calculations of the Gibbs free energy changes for the conditions of the experiment (T = 60 °C, pH = 6.8, *f*(CO_2_) = 0.2 bar, [Ac] = 0.001 mol) revealed that siderite is unstable and could be oxidised by anaerobic thermophiles through the following reaction:

$${\text{12FeCO}}_{{3}} + {\text{2H}}_{{2}} {\text{O }} = {\text{ CH}}_{{3}} {\text{COO}}^{ - } + {\text{ 4Fe}}_{{3}} {\text{O}}_{{4}} + {1}0{\text{CO}}_{{2}} + {\text{H}}^{ + } \Delta G_{r}^\circ = \, + {73}.{\text{1 kJ}}/{\text{mol}}; \, \Delta G_{r}^{exp} = \, - {33}.{9}0{\text{ kJ}}/{\text{mol}}$$

**Figure 4:**
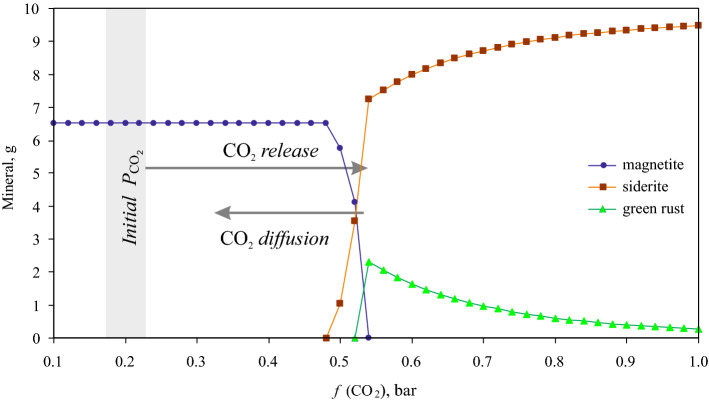
Thermodynamic calculations of equilibrium mineral associations at *f*(CO_2_) from 0.1 to 1.0 bar made for experimental conditions. Initial CO_2_ fugacity (0.2 bar) changes in the layer of siderite which is not stable. Carbon dioxide released by reaction (1) can induce a local increase of CO_2_. At a carbon dioxide fugacity more than 0.52 bar siderite becomes stable again.

**Figure 5:**
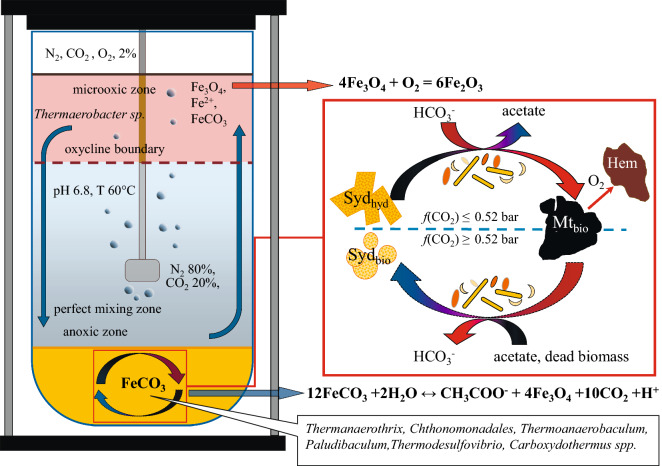
Schematic representation of the proposed processes in the bioreactor culture*.* *Sid*_*hyd*_ hydrothermal siderite, *Sid*_*bio*_ metabolically induced siderite, *Mt*_*bio*_ metabolically induced magnetite, *Hem* hematite, *red* *square* anaerobic iron cycle driven by the thermophilic microbial consortium at the bottom of the bioreactor (in the mineral sludge).

The equilibrium value of the CO_2_ fugacity (0.52 bar) allowed the coexistence of siderite and magnetite but restricted further oxidation. At higher CO_2_ fugacity, magnetite becomes unstable (Fig. 4). Carbon dioxide released in this reaction (1) can induce a local increase in CO_2_ fugacity inside the unmixed mineral sediment and, by doing so, initiate a reverse reaction of magnetite reduction to siderite. This reverse reaction can be performed by the organotrophic iron-reducing microorganisms present in the consortium, in particular those of the genera *Carboxydothermus*, *Thermodesulfovibrio*, *Thermoanaerobaculum* or *Paludibaculum*, which could use newly formed acetate or biomass of autotrophs as the electron donor. The newly formed siderite in the bioreactor crystallised in the form of spherical aggregates identical to those we observed in the enrichment culture (Supplementary Fig. S2c) and was quite different from the original hydrothermal siderite particles (Figs. 2, 3). Such a morphology is fairly common for sedimentary siderite precipitated from saturated solutions^8^, in the reaction of glucose with ferrihydrite at pressure and temperature typical to diagenesis^3^, or formed by dissimilatory iron reducers^14,32,47^. Moreover, the parameters of Mössbauer spectra of siderite obtained in our experiments were identical to those reported previously for siderite produced by the thermophilic bacterium *Thermincola* *ferriacetica* in the course of ferrihydrite reduction^48^. Given these facts and the thermodynamic calculations, we propose the formation of metabolically induced siderite as the most probable process closing the iron cycle performed by the thermophilic microbial consortium (Fig. 5). The content of the newly formed siderite increased during the incubation, while the relative content of magnetite remained practically constant (Table 1; Supplementary Fig. S5), meaning the microbial consortium acted as a conveyor transforming hydrothermal siderite to new siderite with magnetite and acetate as intermediate products. The diffusion rate of carbon dioxide in the mineral and liquid phases and the metastability of siderite seems to serve as the master switch of this cycle, turning on or off the formation of magnetite (Fig. 5).

The precipitation of hematite that occurred in the upper part of the bioreactor at the water–gas interface (Supplementary Fig. S8) was caused by oxidation of magnetite and siderite particles uplifted to the microoxic zone of the bioreactor during the sampling procedure (Fig. 5). Hematite, is a thermodynamically stable phase, accumulated as a secondary mineral during the experiment and, most likely, did not participate in the iron cycle driven by the anaerobic microbial consortium due to its low bioavailability as electron acceptor^19,49^.

The anaerobic microbial cycle described here has important geological implications for the period of BIF deposition. In contrast to photoferrotrophic or nitrate-dependent Fe(II)-oxidising processes, it is not limited by the availability of electron acceptors. It is widely recognised that the primary source of iron minerals in BIF was ferrous iron carried by hydrothermal fluid^5–8^ and that the CO_2_ partial pressure and carbonate content never dropped below their modern levels throughout the Earth’s geological history^50^. Thus, the presumed conditions of early Earth could favour the evolution of anaerobic Fe(II)-oxidising microorganisms, which can use CO_2_ as the only electron acceptor and carbon source via acetogenesis which was considered to be the most ancient pathway^34,51^. In our experiments, the selected microbial consortium was able to grow without allochthonous organic matter by utilising the energy of siderite oxidation, with CO_2_ as the electron acceptor and acetate as the reduced product. The rate of siderite transformation calculated from Mössbauer data (Table 1) was high: 0.5% hydrothermal siderite per day was transformed into magnetite, hematite and metabolically induced siderite. The organic matter produced in this process, i.e., acetate and cell biomass, served as the electron donors for the iron reducers, thus making further biogenic transformation of magnetite possible. The cycle resulted in the accumulation of organic matter [HAWK analysis indicated accumulation of 123 mg TOC (Supplementary Table S5)] and of a mixture of oxidised iron minerals, magnetite and secondarily formed hematite. Such a light- and oxygen-independent biological cycle of iron—from siderite to novel siderite—could have served as the driving force in the ancient biosphere, much like the cycle of oxygen (i.e., photosynthesis and respiration reactions) has served as the central biogeochemical driving force for major processes in the biosphere since the Great Oxygenation Event. Notably, the anaerobic biogeochemical iron cycle could also play a significant role in certain parts of the modern biosphere—in deep subsurface and other extreme environments devoid of light and oxygen or its derivatives but rich in redox-active iron minerals.

## Conclusions

Our experiments clearly show the capability of an autotrophic thermophilic microbial consortium to perform anaerobic oxidation of hydrothermal siderite. We demonstrate that the process of anaerobic iron oxidation coupled with acetate production, which has been previously shown for alkaliphiles^17,27,28^, is likely to have wider distribution and could be performed by microbial communities inhabiting pH-neutral thermal environments.

Thermodynamic calculations performed for our experimental conditions confirm that the communities of thermophilic bacteria can gain energy in the course of a short cycle of iron minerals redox transformations, utilising CO_2_ as the electron acceptor and the primary carbon source. Growth of thermophilic bacteria based on two low-energy reactions of siderite oxidation and magnetite reduction makes the fugacity of CO_2_ a master switch of this short iron cycle, either favouring, or hampering magnetite formation.

The described iron cycle sustains primary biomass production from CO_2_ by chemosynthesis, thus representing one of the processes that could drive biogeochemical cycles of carbon and iron in the early Earth’s biosphere as well as in modern deep subsurface biosphere. The main benefit of this process in comparison with previously known mechanisms of microbial anaerobic iron oxidation is that the process is not limited by the availability of electron donors and acceptors. The high availability of both reduced iron and CO_2_ compensate for the low energy efficiency of carbonate-dependent siderite oxidation and facilitate magnetite reduction, making these reactions possible driving forces in the ancient biosphere.

The accumulation of the main BIF iron mineral association, i.e. hematite, magnetite and metabolically induced siderite, could arise from the difference in microbial siderite oxidation and magnetite reduction rates as well as from the low hematite bioavailability.

## Materials and methods

### Characteristic of the sampling site, sampling and obtaining of the enrichment culture

Samples of water and sediment were taken from the Solnechny (Sun) hot spring in the Uzon Caldera (54° 29.5632  N/159° 59.3164 E, 52 °C, pH 6.1), Kamchatka Peninsula, Russia, in 2015. The spring discharges at the bottom of a thermally heated swamp. The bottom of the spring is shaped like a funnel, is covered with a layer of red to orange Fe-containing deposits and is characterized by active CO_2_ emanations from the central hole^35^. The temperature and pH levels in the spring have remained fairly stable over several years of observations; the *E*_*h*_ value is negative and comprises − 34 mV (vs standard hydrogen electrode, SHE) at a depth of 0.5 m and around zero on the water surface where it is influenced by atmospheric precipitations. The sample was taken at a depth of 10 sm below the sediment surface and transported to the laboratory in sterile anaerobic 50 ml flasks.

### Characteristics of siderite

Selected grains of hydrothermal siderite FeCO_3_ (Bakal deposit, Ural, Russia) were crushed into powder (< 100 µm particle size) in an agate mortar. The purity of the siderite was confirmed by X-ray diffraction that revealed no detectable impurities from secondary minerals (Supplementary Fig. S6). The absence of Fe(III) in siderite was confirmed by Mössbauer spectroscopy.

### Cultivation techniques

The standard anaerobic modified Pfennig medium (mP) was used for the enrichment (g l^−1^): 0.33 KCl, 0.33 MgCl_2_ × 6H_2_O, 0.11 CaCl_2_, 0.33 NH_4_Cl, 0.33 KH_2_PO_4_. After boiling, the medium was cooled under a flow of N_2_/CO_2_ (80/20 v/v) and simultaneously supplied with 0.7 g l^−1^ of NaHCO_3_, 1 ml l^−1^ of trace element solution, 1 ml l^−1^ of vitamin solution^23^. The sediment and water samples from the Solnechny hot spring (2 ml) were added to the 30 ml of medium that contained 10 g l^−1^ of hydrothermal siderite powder, and the mixture incubated in 50-ml flasks under N_2_/CO_2_ (80:20, v/v) at 60 °C and pH 6.8–7.0. The resulting enrichment culture was transferred (5% v/v) to a fresh medium each time the colour of the mineral phase changed from orange to grey. After five transfers, the enrichment was further processed in Hungate tubes using the tenfold dilution method, and the last positive dilution (10^–6^) was transferred into 50-ml flasks five times more. Control flasks were not inoculated but were incubated at the same conditions.

A stable, anaerobic thermophilic consortium that was actively transforming hydrothermal siderite, was transferred into a bioreactor set up to simulate Proterozoic environmental conditions: 180 ml inoculum (6% v/v) was anaerobically transferred to a benchtop bioreactor Labfors 4 (Infors HT, Switzerland) pre-filled with 3 L sterile medium and 300 g hydrothermal siderite powder. The bioreactor was started in batch mode with the temperature and pH controlled, and incubation was carried out over 56 days using the following mixing regime: the bottom of the reactor, a layer of a solid mineral phase (ca. 300 ml volume) was left almost unstirred. The water column over the solid phase was intermittently bubbled with a N_2_/CO_2_ gas mixture (80/20 v/v) and subjected to an exact mixing model with Rushton impellers mounted to the middle of the shaft operated at 50 rpm. Over the water column, a small gas headspace (0.6 l), containing a N_2_/CO_2_/O_2_ gas mixture (78/20/2% v/v), was left with a controlled gas outflow in order to prevent gas overpressure. The volumetric ratio between the solid, liquid and gas phases of the bioreactor culture was ca. 1:9:2 (v/v). To monitor microbial growth, acetate production and siderite transformation, subsamples were taken every 7 days during the incubation period. Before each sampling, the impellers were set to 450 rpm for 30 min to provide uniform distribution of the culture components throughout its volume. Outflow gas was regularly sampled and analysed via a flow-through autosampler of a gas chromatograph (see below) connected to the exit gas cooler of the bioreactor by oxygen-tight tubing.

Uninoculated controls were incubated under the same conditions for the same duration of time in 0.5 l bottles with the same mineral/liquid/gas phases volumetric ratio (1:9:2). Sampling of gas and liquid phases was performed at the same intervals after the control bottles were thoroughly shaken by hand.

### Analytical methods

Microbial growth was monitored by direct cell counting with a phase contrast light microscope (Olympus CX41RF, Japan). Possible organic products of growth (volatile fatty acids and alcohols) were assayed using a Stayer HPLC chromatograph (Aquilon, Russia), as described previously^16^. The gas phase was analysed using a ‘3700’ custom-modified gas chromatograph (ZIOC RAS Special Design Tech. Dept., Russia), as described previously^23^.

pH and *E*_*h*_ parameters of the bioreactor culture were monitored automatically using combination electrodes 405 and Pt4805, respectively (type DPAS-SC-K8S/325 by Mettler Toledo), with adjustable immersion depth. For routine monitoring, both electrode tips were immersed down to the middle of the culture’s water column. At the end of incubation, the Redox electrode was lifted to the top layer of the culture to reveal the proposed oxycline boundary zone. pH and *E*_*h*_ parameters of the cultures and controls that were incubated in flasks were measured in subsamples taken aseptically immediately after their collection,using a Seven2Go Pro pH/mV portable meter equipped with InLab series pH and Redox combination electrodes (all from Mettler Toledo).

Soluble and HCl-extractable iron species were determined from culture subsamples taken in diluted (0.6 N) HCl. Insoluble material was separated from HCl extracts by centrifugation at 12 kg for 10 min on an Eppendorf table top centrifuge. Clean extract was further processed for colorimetric detection of Fe^2+^ with ferrozine and Fe^3+^ with potassium thiocyanate, as previously described^52^.

The morphology of mineral samples was examined by SEM using a TESCAN VEGA 3 LMU device with an INCA Energy 350/X-max 80 energy-dispersive (EDS) analysis system (OXFORD Instruments NanoAnalysis, UK). Patterns were pre-fixed with double-sided carbon tape and triply coated with Au.

Mineral compositions were determined by X-ray diffraction analysis on a Panalytical Empyrean diffractometer using a copper anode (λ = 1.54 Å, I = 40 mA, U = 40 kV).

The Fe^2+^/Fe^3+^ ratio was determined by ^57^Fe Mössbauer spectroscopy. This method allows for the determination and quantification of different atomic environments, magnetic states, chemical states and transformations of iron-containing compounds^53^. All Mössbauer measurements were carried out at room temperature using an MS-1101 Em spectrometer operating in the constant acceleration mode, with ^57^Co source in Rh matrix. To reduce the texture effect, Mössbauer measurements were performed at the ''magic angle'' (the absorber placed at an angle ϑ = 54.74° to γ-ray direction) by placing the siderite powder samples in a paraffin cone. The calibration was constructed relative to α-Fe, and spectra were fitted with SpectrRelax software^54^. When the relative content of magnetite was low, sextets that correspond to iron atoms in the A and B positions of magnetite are not resolved. In this case, a model consisting of one sextet with broadened lines with hyperfine parameters equal to the average values of parameters typical for iron atoms in the A and B positions was used for spectrum processing.

E-4 spectrometer (9.2 GHz, Varian) was used to measure the EPR at room temperature. Small grains of siderite powder samples were placed at the bottom of a narrow quartz ampoule and fixed by paraffin. The spectra were recorded using a modulation frequency of 100 kHz and microwave power of 10 mW.

For the identification of organic carbon in control and experimental siderite samples, Rock–Eval pyrolysis method^33^ on HAWK Resource Workstation (Wildcat Technologies, USA) were used. The main measured organic parameters were S1 (free oil)—the amount of thermally freed hydrocarbons (C_8_ ÷ C_15+_) in the sample obtained after heating the sample to 300 ^0^C; S2 (kerogen yield)—the amount of hydrocarbons generated through thermal (300 ÷ 650 °C, heating 25 °C min^−1^) cracking of non-volatile organic matter (kerogen); and S3 (organic carbon dioxide yield)—the amount of CO_2_ produced during pyrolysis of kerogen, corresponding to the organic carbon dioxide yield of the rock and measured by infrared (IR) cell. TOC (wt. %) was calculated as described previously^33^ based on the measured parameters.

### 16S rRNA gene amplification and sequencing

After the culture samples were subjected to cell disruption by bead-beater and proteinase k treatment, DNA was extracted by routine phenol–chloroform method with ethanol precipitation. The V3–V4 region of 16S rRNA genes were amplified and libraries were prepared as previously described^55^. Sequencing of the libraries was performed with Illumina MiSeq platform using MiSeq Reagent Kit v3 (600 cycles). Most of the data analysis, including quality trimming, demultiplexing, taxonomic assignments and core diversity analyses were performed with QIIME^56^ and SILVA online data analysis service^57^. All sequencing data were entered into NCBI BioProject PRJNA626446. Basic statistics of the results obtained from the sequencing of the V3-V4 regions of the 16S rRNA gene are presented in the Supplementary Table S6.

### Thermodynamic calculations

Equilibrium state calculations for the system (H–C–O–Na–Fe) were carried out using the HCh software^58,59^. To calculate the equilibrium state, the minimum free energy of the system was determined. The thermodynamic model was analysed to determine the proportions of the phases in the experiment at a variable CO_2_ partial pressure. These calculations show phase associations and solution compositions that correspond to thermodynamic equilibrium. It should be noted that such equilibrium was not reached during the experiment. The calculations were used to determine in which direction processes were moving.

Changes in Gibbs free energy of reactions were calculated using the HCh software, and the thermodynamic properties of components were taken from the Unitherm database^59^. The values for the standard state (Δ*G*°_r_) were recalculated for real pH values, and bicarbonate activity and free energy for reactions with experimental component activities (Δ*G*_*r*_) were received. These values can be used to determine the direction of the process. Thermodynamic properties for green rust with composition FeCO_3_ × 3Fe(OH)_2_ × 2Fe(OH)_3_ × 2H_2_O were taken from Drissi et al.^60^.

## Supplementary Information

Supplementary information.
