# Supplementary material for: Siderite-based anaerobic iron cycle driven by autotrophic thermophilic microbial consortium
Source: Sci Rep. 2020 Dec 10;10:21661. doi: 10.1038/s41598-020-78605-7 (PMC7729950; doi:10.1038/s41598-020-78605-7)

Siderite-based anaerobic iron cycle driven by autotrophic thermophilic microbial consortium

Daria G. Zavarzina[1], Tatiana V. Kochetkova[1], Nataliya I. Chistyakova[2], Maria A. Gracheva[2,3], Angelina V. Antonova[2], Alexander Yu. Merkel[1], Anna A. Perevalova[1], Michail S. Chernov[4], Yury A. Koksharov[2], Elizaveta A. Bonch-Osmolovskaya[1,5], Sergey N. Gavrilov[1], Andrey Yu. Bychkov[4]

SUPPLEMENTARY MATERIALS

Suppl. Table 1. Hyperfine parameters of Mössbauer subspectra of a solid phase sample from the stable siderite-oxidizing anaerobic thermophilic microbial consortium obtained after 10 consequent transfers of primary enrichment on the medium with hydrothermal siderite. *I* – relative intensity, *δ* – isomer shift, *ε* – quadrupole shift.

Phase	*I*, %	*δ*, mm/s	*ε*, mm/s
hydrothermal siderite (D_1)	89.6±2.2	1.22±0.01	0.88±0.01
green rust Fe^{2+} (D_2)	5.3±1.1	1.22±0.06	1.32±0.08
green rust Fe^{3+} (D_3)	5.1±0.8	0.31±0.08	0.22±0.03

Data given with standard deviation

Suppl. Table 2. Hyperfine parameters of the Mössbauer subspectrum of the solid phase of the bioreactor culture sampled at the end of incubation. I – relative intensity, δ – isomer shift, ε – quadrupole shift, H – hyperfine magnetic field.

Phase	*I,* %	*δ,* mm/s	*ε,* mm/s	*H*, kOe
hydrothermal siderite (D_1)	70.0±3.0	1.23±0.01	0.88±0.01	–
metabolically induced siderite (D_2)	23.0±3.0	1.19±0.01	0.92±0.01	–
magnetite (S)	7.0 ±4.0	0.36±0.03	-0.11±0.03	488±2

Data given with standard deviation

Suppl. Table 3. Hyperfine parameters of the Mössbauer subspectrum of the solid phase collected from bioreactor walls in the oxycline boundary zone of the culture at the end of incubation. I – relative intensity, δ – isomer shift, ε – quadrupole shift, H – hyperfine magnetic field.

Phase	*I,* %	*δ,* mm/s	*ε,* mm/s	*H*, kOe
hydrothermal siderite (D_1)	52.9±2.9	1.23±0.01	0.88±0.01	–
green rust (D_2)	17.5±2.8	1.19±0.01	0.95±0.01	–
Fe(III)-containing phase (D_3)	10.6±0.5	0.36±0.01	0.37±0.01	–
hematite (S)	18.9±0.6	0.36±0.01	-0.09±0.01	515±2

Data given with standard deviation

Suppl. Table 4. Data of EDS analysis of spectra obtained from hydrothermal siderite and spheric-shaped particles indicated on the Figure 2. In the Table are presented only those elements which content exceed 1 wt.% (weight percent); σ - population standard deviation.

	elements	O		C		Fe		Si		Al	
	spectra	wt.%	σ	wt.%	σ	wt.%	σ	wt.%	σ	wt.%	σ
hydrothermal siderite particles	EDS1	59.1	1.5	16.2	1.2	12.1	0.4	5.5	0.2	1.6	0.1
	EDS 3	62.5	0.9	20.6	0.9	14.5	0.3	0	0	0	0
	EDS 5	63.6	3.9	17.6	3.3	10.6	0.9	0	0	0	0
	EDS 9	65.5	4.3	21.9	3.3	7.4	0.7	0	0	0	0
	EDS 11	65.7	2.8	22.6	2.8	8.9	0.6	0	0	0	0
spheric-shaped particles	EDS 2	52.5	2	12.7	1.5	20.8	0.8	0	0	0	0
	EDS 4	60.9	1.4	19	1.1	14.1	0.4	0	0	0	0
	EDS6	64.4	3.8	20.1	3.8	3.4	0.3	2.1	0.2	0	0
	EDS7	64.4	3.8	20.1	3.8	3.4	0.3	2.1	0.2	0	0
	EDS 8	65.9	3.8	18.5	2.9	5.4	0.5	0	0	0	0
	EDS10	66.2	3.8	19.9	2.9	4.8	0.4	0	0	0	0

	elements	Na		Cl		P		N	
	spectra	wt.%	σ	wt.%	σ	wt.%	σ	wt.%	σ
hydrothermal siderite particles	EDS1	1.2	0.1	1.2	0	0	0	0	0
	EDS 3	0	0	0	0	0	0	0	0
	EDS 5	0	0	0	0	1.1	0.2	0	0
	EDS 9	0	0	0	0	1.5	0.2	0	0
	EDS 11	0	0	0	0	0	0	0	0
spheric-shaped particles	EDS 2	0	0	1.5	0.1	2.9	0.1	0	0
	EDS 4	0	0	0	0	1.3	0.1	0	0
	EDS6	0	0	0	0	3.2	0.3	0	0
	EDS7	0	0	0	0	3.2	0.3	0	0
	EDS 8	0	0	0	0	2.2	0.2	3.2	3
	EDS10	0	0	0	0	2.1	0.2	2.1	3.1

Suppl. Table 5. Data of HAWL analysis performed after the end of incubation for mineral phases from bioreactor and control.

**Sample**	**S1**	**S2**	**S3**	**TOC**
	Free oil, mg HC/g	Kerogen yield, mg HC/g	Organic carbon dioxide yield, $mgCO_2/g$	Total Organic Carbon, wt. %
control	0.00	0.02	4.74	**0.002**
experiment	0.09	0.43	4.12	**0.043**

Suppl. Table 6. Basic statistics of the obtained results of sequencing of the V3-V4 region of the 16S rRNA gene.

Day number (SRA link)	Total number of sequences	Total number of OTUs	Chao1	Good's coverage	Shannon index
14 (V2F3R4)	192942	142	242	1.00	0.347
28 (V2F3R3)	57855	128	189	1.00	0.203
42 (V2F2R6)	53010	124	243	1.00	0.964
56 (V2F2R5)	50236	209	394	1.00	1.032

Suppl. Figure 1. Change of the color of the mineral phase, supplied as hydrothermal siderite, in the enrichment culture obtained after 10-fold dilution procedure. Left Hungate tube – sterile control; right Hungate tube – the last positive dilution (10^{-6}) of the primary enrichment.

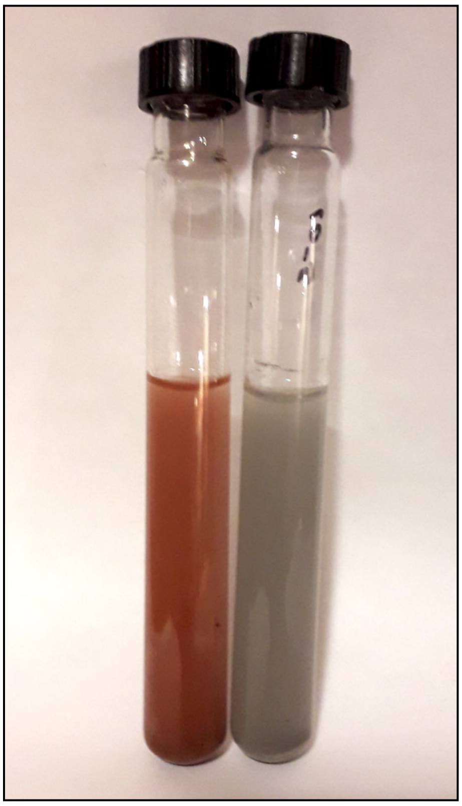

Suppl. Figure 2. Mössbauer spectra of the solid phase sample from the microbial consortium obtained after 10 consequent transfers of the primary enrichment on the medium with hydrothermal siderite. Mössbauer spectra measured at room temperature (a); at the "magic angle" θ = 54.7° (b); For the comparison, the Mössbauer spectra of a sterile control measured at room temperature (c); at the "magic angle" θ = 54.7° (d). In the figure D_1 corresponds to Fe^{2+} ions in the structure of initial siderite, D_2 – Fe^{2+} ions in the structure of green rust, D_3 – Fe^{3+} ions in the structure of green rust.

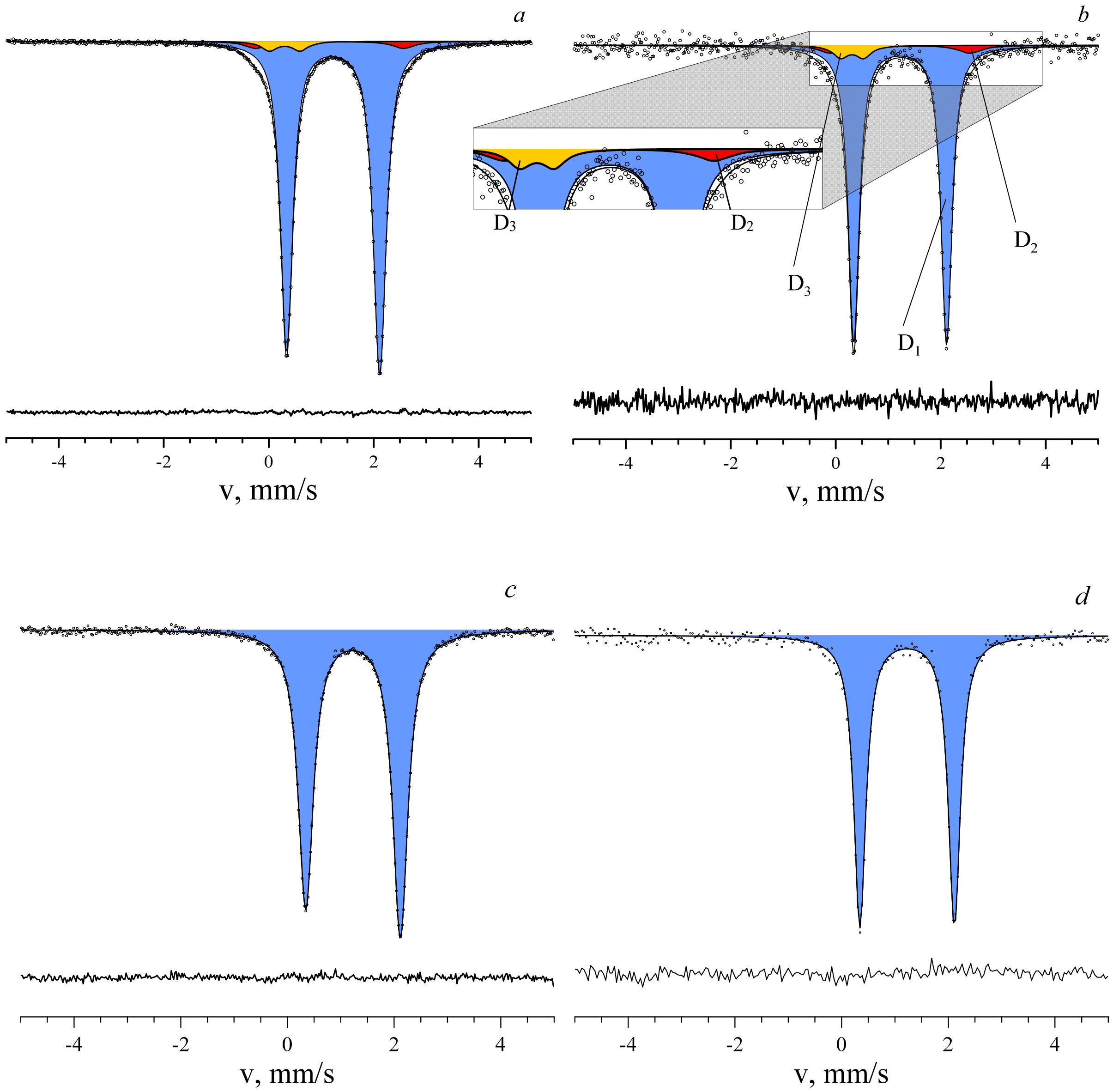

Suppl. Figure 3. SEM micrographs of the solid phase sample from the microbial consortium obtained after 10 consequent transfers of the primary enrichment on the medium with hydrothermal siderite. (a) intact hydrothermal siderite in the sterile control; (b, c) different magnifications of the mineral phase of the grown culture: particles of hydrothermal siderite with green rust crystals formed on their surface (white arrows) and dense globules of another mineral phase formed separately (blue arrows, further identified as biogenic siderite); (d) general view of lamellae of green rust; (e, f) tabular crystals of green rust fastened in a mutually perpendicular direction.

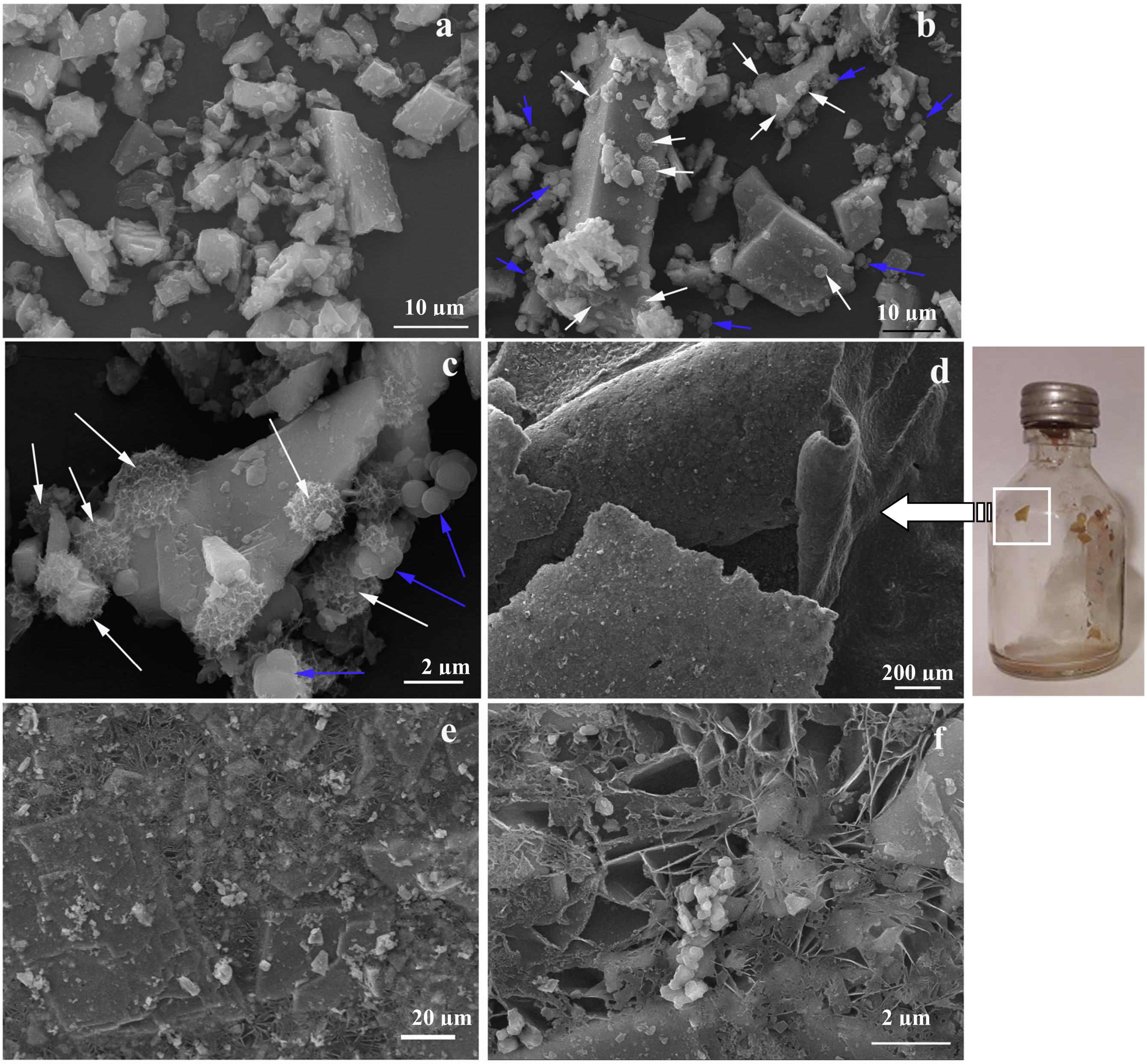

Suppl. Figure 4. Phylogenetic composition (%) of the microbial consortium obtained after 10 consequent transfers of the primary enrichment on the medium with hydrothermal siderite.

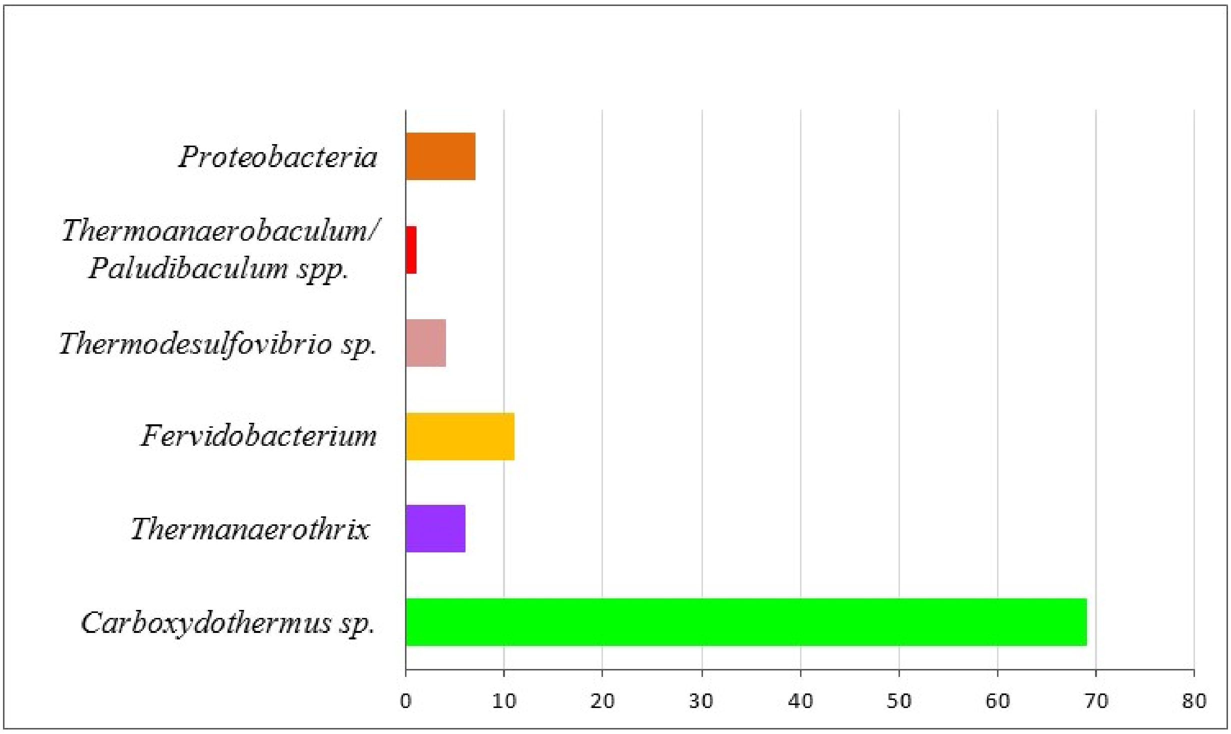

Suppl. Figure 5. Changes of the mineral phase composition of the bioreactor culture within its incubation period.

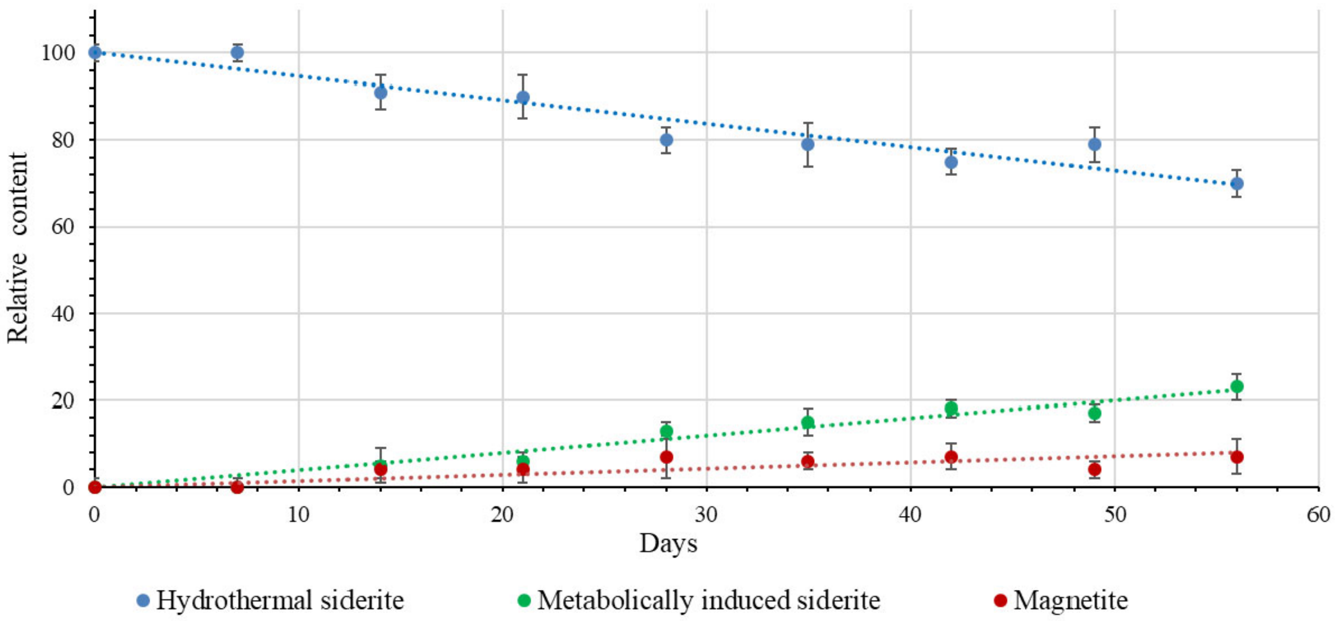

Suppl. Figure 6. X-ray pattern of the mineral phase of the bioreactor culture at the end of incubation.

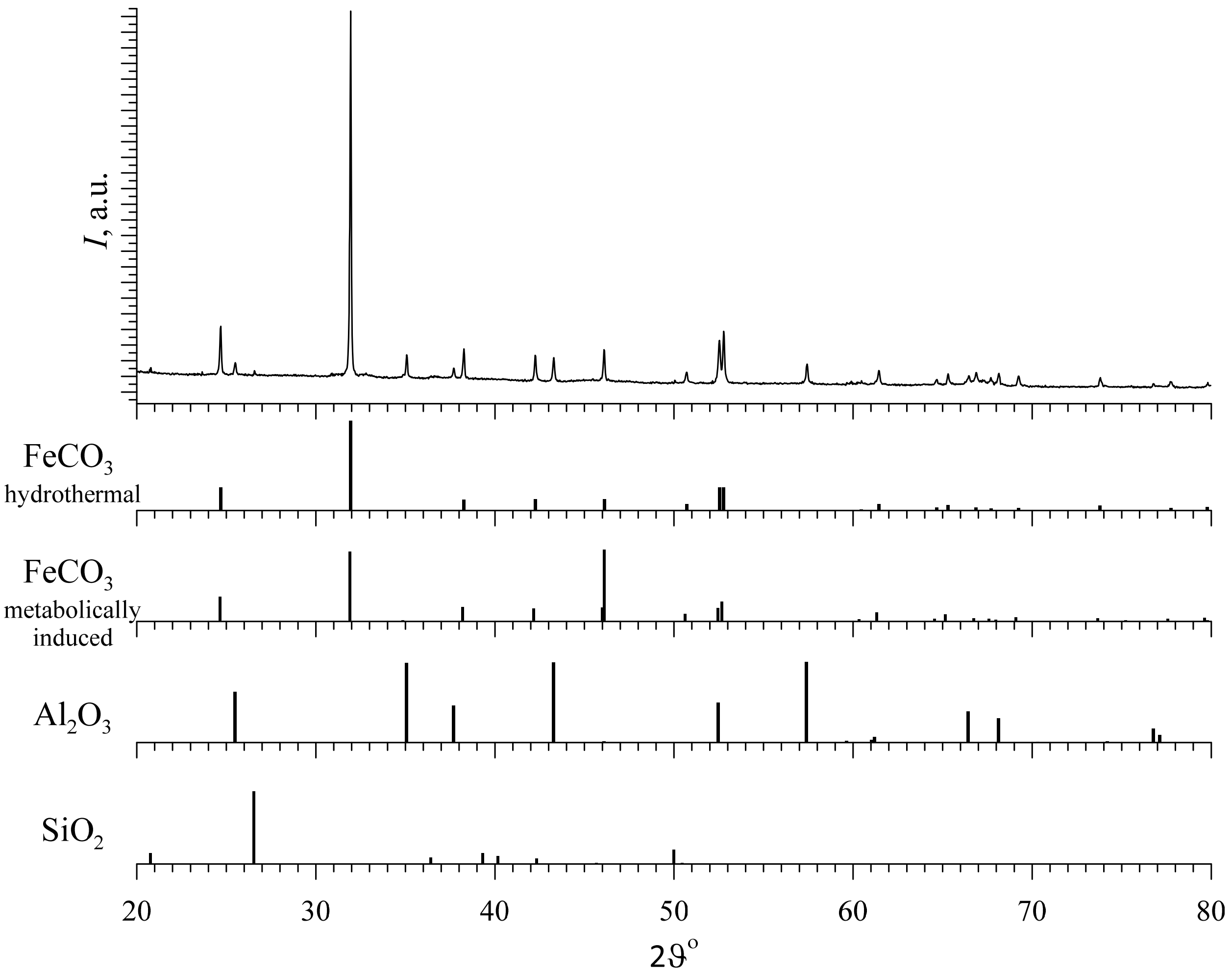

Suppl. Figure 7. EPR spectra of solid phases of the bioreactor culture (red) and the sterile control (black) sampled after the end of incubation.

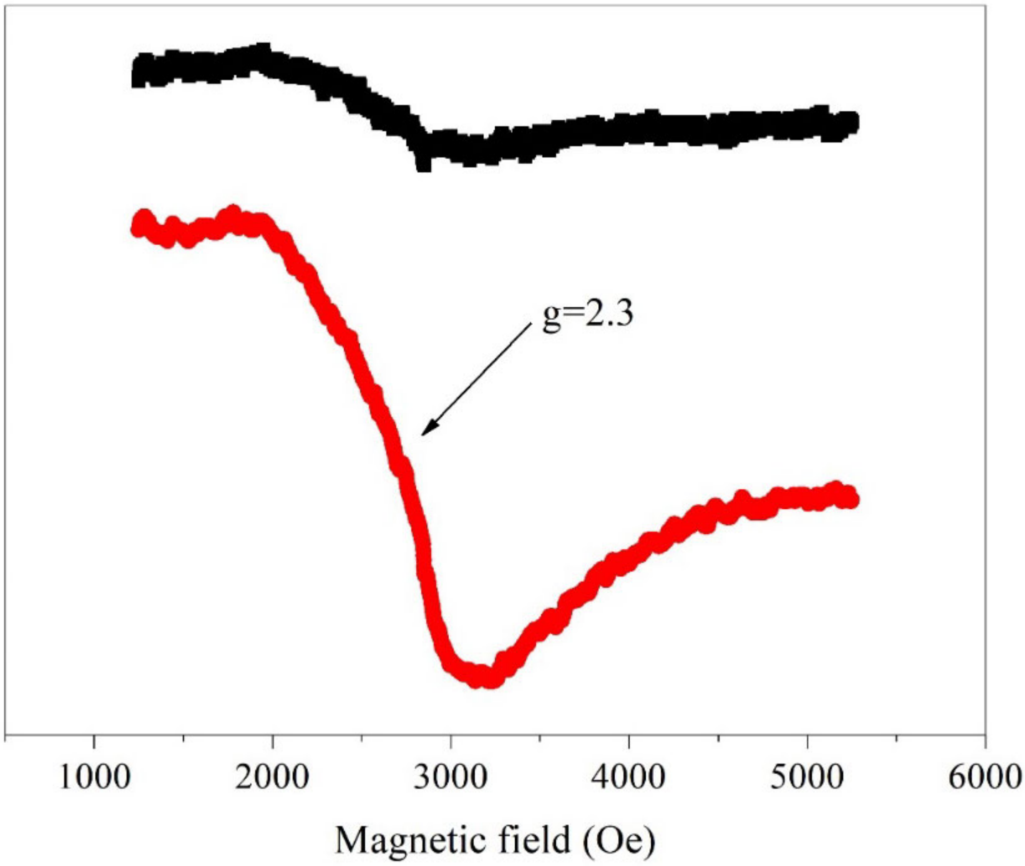

Suppl. Figure 8. Photos of empty bioreactor before the experiment (left) and after the end of incubation (right) with the mineral phase precipitated on the upper part of bioreactor walls at the oxycline boundary zone of the culture (reddish coating on the right photo).

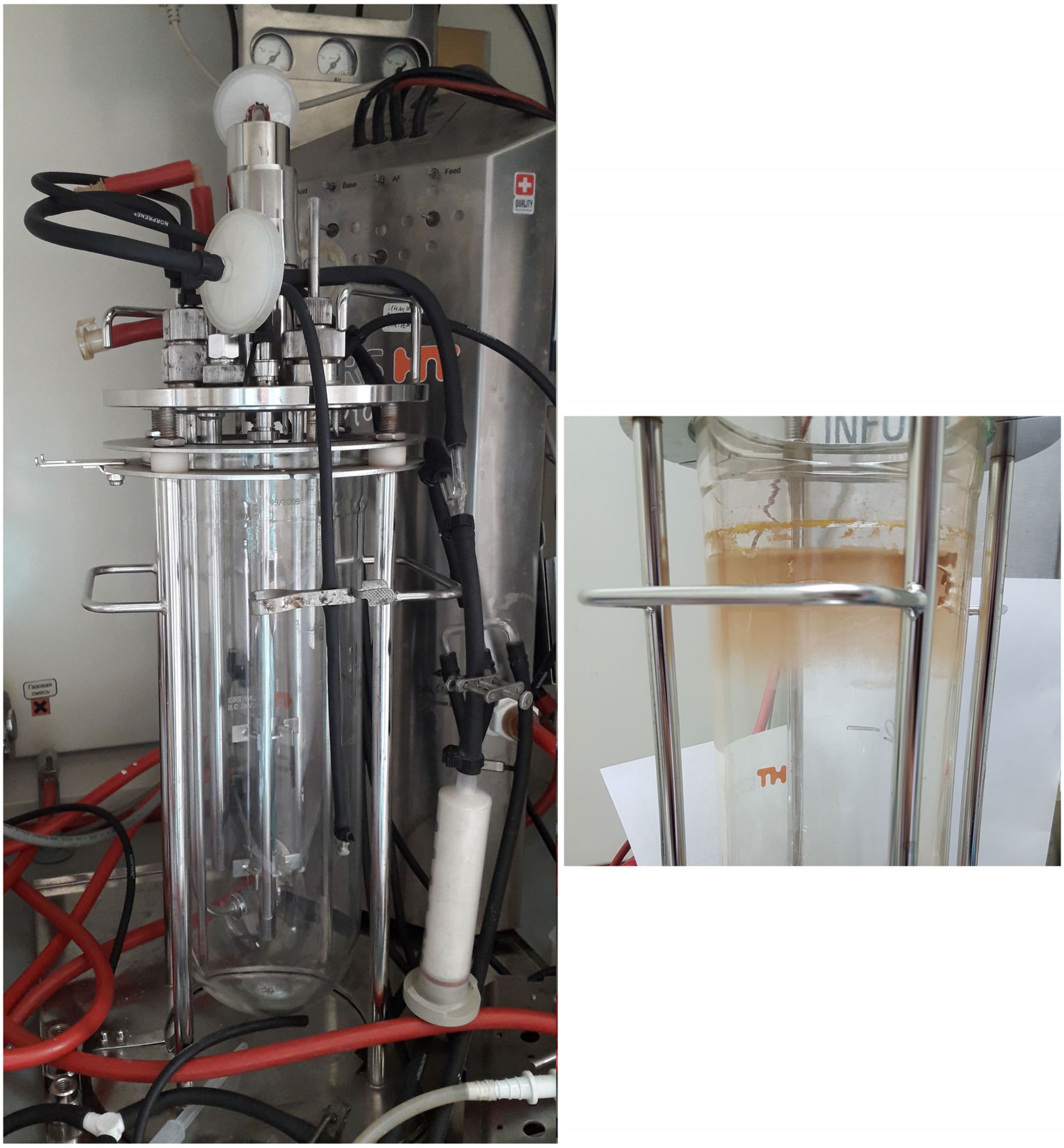

Suppl. Figure 9. Mössbauer spectrum of a sample of the mineral phase precipitated on the upper part of bioreactor walls at the oxycline boundary zone of the culture. In the figure D_1 corresponds to Fe^{2+} ions in the structure of hydrothermal siderite, D_2 – Fe^{2+} ions in the structure of green rust, D_3 – Fe^{3+} ions in the structure of Fe(III)-containing phase, S – microparticles of hematite.

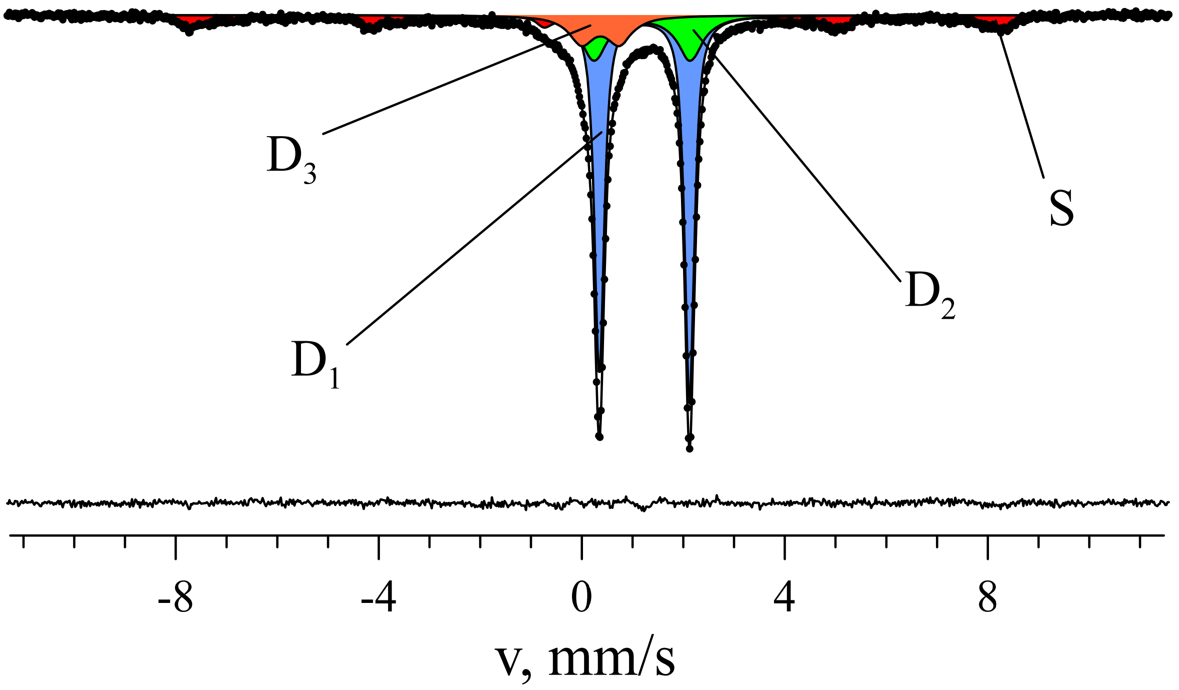

Supplement: Supplementary file 1 — Supplementary information. [file 41598_2020_78605_MOESM1_ESM.pdf]
